# *DLX* Genes: Roles in Development and Cancer

**DOI:** 10.3390/cancers13123005

**Published:** 2021-06-15

**Authors:** Yinfei Tan, Joseph R. Testa

**Affiliations:** 1 Genomics Facility, Fox Chase Cancer Center, Philadelphia, PA 19111, USA; 2 Cancer Signaling and Epigenetics Program, Fox Chase Cancer Center, Philadelphia, PA 19111, USA

**Keywords:** homeobox genes, HOX, DLX, hematopoiesis, development, cancer, lymphoma, leukemia, Aka

## Abstract

**Simple Summary:**

*DLX* homeobox family genes encode transcription factors that have indispensable roles in embryonic and postnatal development. These genes are critically linked to the morphogenesis of craniofacial structures, branchial arches, forebrain, and sensory organs. *DLX* genes are also involved in postnatal homeostasis, particularly hematopoiesis and, when dysregulated, oncogenesis. *DLX1/2*, *DLX3/4*, and *DLX5/6* exist as bigenes on different chromosomes, sharing intergenic enhancers between gene pairs, which allows orchestrated spatiotemporal expression. Genomic alterations of human *DLX* gene enhancers or coding sequences result in congenital disorders such as split-hand/foot malformation. Aberrant postnatal expression of *DLX* genes is associated with hematological malignancies, including leukemias and lymphomas. In several mouse models of T-cell lymphoma, *Dlx5* has been shown to act as an oncogene by cooperating with activated Akt, *Notch1/3*, and/or Wnt to drive tumor formation. In humans, *DLX5* is aberrantly expressed in lung and ovarian carcinomas and holds promise as a therapeutic target.

**Abstract:**

Homeobox genes control body patterning and cell-fate decisions during development. The homeobox genes consist of many families, only some of which have been investigated regarding a possible role in tumorigenesis. Dysregulation of *HOX* family genes have been widely implicated in cancer etiology. *DLX* homeobox genes, which belong to the NK-like family, exert dual roles in development and cancer. The *DLX* genes are the key transcription factors involved in regulating the development of craniofacial structures in vertebrates. The three *DLX* bigenes have overlapping expression in the branchial arches. Disruption of *DLX* function has destructive consequences in organogenesis and is associated with certain congenital disorders in humans. The role of *DLX* genes in oncogenesis is only beginning to emerge. DLX2 diminishes cellular senescence by regulating p53 function, whereas DLX4 has been associated with metastasis in breast cancer. In human ovarian cancer cells, DLX5 is essential for regulating AKT signaling, thereby promoting cell proliferation and survival. We previously implicated *Dlx5* as an oncogene in murine T-cell lymphoma driven by a constitutively active form of *Akt2*. In this mouse model, overexpression of *Dlx5* was caused by a chromosomal rearrangement that juxtaposed the Tcr-beta promoter region near the *Dlx5* locus. Moreover, transgenic mice overexpressing *Dlx5,* specifically in immature T-cells, develop spontaneous thymic lymphomas. Oncogenesis in this mouse model involves binding of Dlx5 to the *Notch1* and *Notch3* gene loci to activate their transcription. Dlx5 also cooperates with Akt signaling to accelerate lymphomagenesis by activating Wnt signaling. We also discuss the fact that human DLX5 is aberrantly expressed in several human malignancies.

## 1. Introduction

Homeobox genes were discovered more than three decades ago. They include a large group of genes that are essential in the development of multicellular organisms of the Metazoan division of the animal kingdom [1]. Cancer is often deemed as development gone awry. While some homeobox genes have oncogenic functions, others exert an opposite role. Homeobox genes have been categorized into 11 gene classes, which have been further categorized into many families during evolution [2,3]. The homeobox motif is a 180-bp DNA sequence encoding the homeodomain, which is highly conserved [4]. The protein product contains the homeodomain composed of helices in which helices II and III form helix-turn-helix (HTH) motifs [5]. This structure permits homeodomain proteins to bind to specific DNA consensus sequences and function as transcription factors [6]. *Drosophila* contains clustered NK-related homeobox genes, and these genes possess homeobox sequences of the ANTP class [7]. The vertebrate NK-like homeobox genes have essential roles in development and cancer [8]. The NKL class is subdivided into many gene families, such as *NKX, NANOG, MSX, TLX,* and *DLX* families [9]. While *NKX, TLX,* and *MSX* family genes have been implicated in T-cell acute lymphoblastic leukemia (T-ALL), the role of the *DLX* family has been less well studied in cancer [10].

## 2. Structure and Origin of the *DLX* Gene Clusters

**Figure 1:**
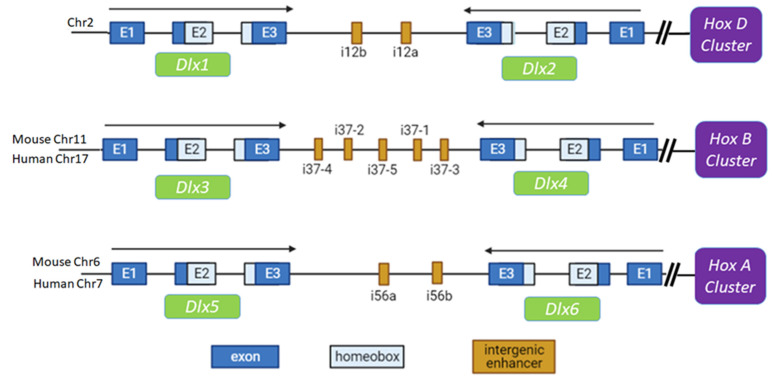
Structure of mouse and human *Dlx* gene family. The *Dlx* genes are comprised of three exons. The homeobox motif (light blue) resides within part of Exon2 and Exon3. The *Dlx1/2* bigene is linked to the *HoxD* cluster on chromosome 2 in both human and mouse, with intergenic enhancer elements i12a and i12b. The *Dlx3/4* bigene is linked to the *HoxB* cluster on mouse chromosome 11, with five intergenic cis elements. The *Dlx5/6* bigene resides along with the *HoxA* cluster on chromosome 6, with i56a/b (or i56i/ii) as the intergenic enhancer.

The *DLX* genes are homologs of *Drosophila* *Distal*-*less* (*Dll*), which was initially found to be specifically expressed in developing limbs [11]. *DLX* genes are now also known to be involved in the morphogenesis of branchial arches, forebrain, and sensory organs [12]. *DLX* genes are comprised of six members in both human and mouse. Interestingly, *DLX1/2*, *DLX3/4,* and *DLX5/6* occur as bigene clusters in the genome on different chromosomes, by sharing intergenic enhancers between two genes, which allow orchestrated spatiotemporal expression [13,14,15]. For example, in mice, *Dlx1* and *Dlx2* are located on chromosome 2 at 42.61 cM and 42.65 cM, respectively, *Dlx3* and *Dlx4* are located on chromosome 11 at 59.01 cM, and *Dlx5* and *Dlx6* are located on chromosome 6 at 2.83 cM. Each of the gene pairs has one gene located in the plus (+) strand and one on the minus (−) strand in a tail-to-tail orientation. The *Dlx1/2* bigene is linked to the *HoxD* cluster on chromosome 2, with intergenic enhancer elements i12a and i12b [16]. The *Dlx3/4* bigene is linked to the *HoxB* cluster on mouse chromosome 11, with five intergenic cis elements [17,18]. The *Dlx5/6* bigene is associated with the *HoxA* cluster on chromosome 6, with i56a/b (or i56i/ii) as the intergenic enhancer [19,20,21] (Figure 1). The intergenic enhancer plays a major role in regulating the expression of *Dlx5/6*. The mi56i-Cre transgenic mice exhibit the same expression pattern of Cre in the R26R strain as those of endogenous *Dlx5* and *Dlx6* in the facial skeleton and specific brain structures in term embryos [22].

The gene pairs orient in a tail-to-tail arrangement with the enhancer in between. It is hypothesized that these three *Dlx* clusters could be duplicates of an ancestral *Dlx* pair, which may have occurred during evolution, with the first gene pair being the result of a tandem gene duplication [23,24]. The expression patterns are similar among these gene pairs, although the *Dlx1/2* enhancer (I12a/b) has little similarity to that of *Dlx5/6* (I5i, I56ii). However, these enhancers have been conserved across species. The *Dlx1/2* enhancer has greater than 75% homology among humans, mice, and zebrafish. Likewise, the *Dlx5/6* enhancer shares more than 80% similarity among these three species [16]. The homeoboxes of the various *Dlx* genes are highly homologous, suggesting they may have somewhat redundant roles.

## 3. *Dlx* Genes in Normal and Aberrant Development

*Dlx* gene family members have indispensable roles in embryonic morphogenesis and postnatal development. Disturbances in the regulatory mechanisms of *Dlx* gene expression or function result in severe consequences. *Dlx1* and *Dlx2* are expressed in the proximal and distal first and second arches. Mutations of *Dlx1* or *Dlx2* alter the proximodistal patterning of the branchial arches, suggesting that *Dlx1* and *Dlx2* have overlapping roles in craniofacial development [25]. Knockout of *Dlx1* or *Dlx2* alone results in abnormalities of the forebrain, and homozygous knockout mice have been shown to die prematurely or at birth [26]. *Dlx1/Dlx2* double mutant mice were found to have defects in the striatal subventricular zone and differentiation of late born striatal neurons [27]. *Dlx* gene members have also been implicated in the morphogenesis of eyes, nose, ears, and teeth. *Dlx1/2* is essential for the formation of the retina, and the absence of *Dlx1/2* results in the apoptosis of retinal ganglion cells and a diminished ganglion cell layer [28], and new born mice were unable to survive with this defect [27]. Interestingly, *Dlx5* and *Dlx6* are also expressed in the developing forebrain. *Dlx1* and *Dlx2* can bind to the intergenic enhancer of *Dlx5/6* and regulates the expression of this bigene. Consequently, expression of *Dlx5/6* was downregulated in *Dlx1/2* double mutant mice [29].

The *Dlx5* gene is expressed in the branchial arches, restricted brain regions, extending appendages, and bones during embryogenesis. *Dlx5* knockout mice die shortly after birth, suffering from craniofacial abnormalities and malformations of the vestibular organ [30]. The craniofacial abnormalities include exencephaly, hypoplastic nasal capsules, and dysmorphic proximal mandibular arch skeleton [12]. In addition to those same defects, *Dlx5/6* double knockout mice exhibit a novel defect in limb development known as split-hand/split-foot malformation (SHFM). However, the spatiotemporal-specific overexpression of the *Dlx5* gene, in the apical ectodermal ridge of *Dlx5/6*-null mice can rescue this limb malformation, indicating that *Dlx5* and *Dlx6* have redundant roles [31]. The craniofacial and limb defects in *Dlx5* knockout and *Dlx5/6* double knockout mice are potentially due to defects in osteoblast maturation [32]. *Dlx5* and *Dlx6* also play a role in the developing vestibular apparatus. *Dlx5/6*-null embryos have otic induction, but cannot form dorsal otic derivatives [33]. The orchestrated expression of *Dlx1/2, Dlx3*, and *Dlx6* are essential for control of enamel formation via direct regulation of ameloblast differentiation [34].

**Figure 2:**
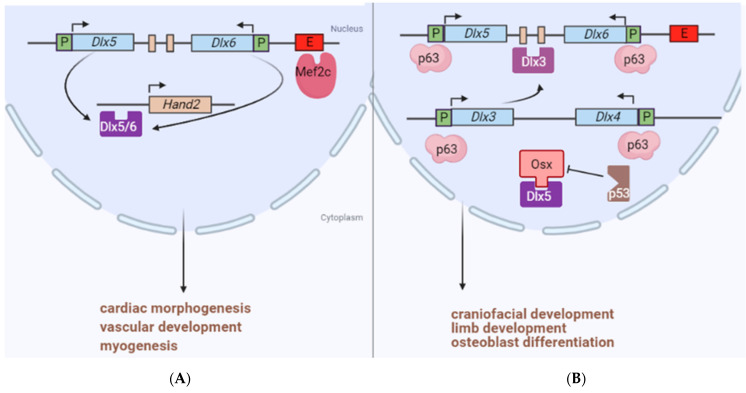
Regulation of Dlx expression. (**A**) Mef2c binds to a novel enhancer (E) outside of the *Dlx5/6* bigene. The Dlx5/6 product activates the *Hand2* gene, which is essential for cardiac morphogenesis, vascular development, and myogenesis; (**B**) P63 transactivates *Dlx3/4* as well as *Dlx5/6* via binding to their promoters. Dlx3 can also regulate *Dlx5/6* expression. Osx functions as a coactivator of Dlx5 in an osteogenic transcriptional network, whereas p53 binds to Osx to inhibit its role.

*Dlx5* and *Dlx6* also play a role in testis development. In fetal Leydig cells, Dlx5 transcriptionally activates the steroidogenic acute regulatory protein gene (*STAR*) via GATA-4, thereby, regulating steroidogenesis [35]. Despite the essential function of *Dlx* genes in craniofacial development, their regulatory mechanism is not well defined. Interestingly, a MADS-box transcription factor *MEF2C*, which is a key to cardiac morphogenesis, vascular development, and myogenesis, controls the expression of *Dlx5/6* in the branchial arches [36] (Figure 2). Moreover, *Tp63* is also involved in limb development, and it can bind to the *Dlx5/6* promoter. *Tp63* knockout mice exhibit severe limb defects with reduced expression of *Dlx* genes [37]. In addition to the intergenic enhancer, there is an enhancer outside of the *DLX5/6* bigene cluster. This new enhancer can drive the expression of a reporter gene in the inner ears and bones of transgenic mice. The deletion of this cis element on human chromosome 7 accounts for a familial syndrome involving hearing loss and craniofacial defects due to reduced expression of DLX5/6 [38]. P63 can also bind to the *Dlx3* promoter [39]. Dlx3 regulates bone formation by controlling the expression of *Dlx5, Dlx6, Runx2,* and *Sp7* [40] (Figure 2). *Dlx2, Dlx5,* and *Dlx6* are expressed most strongly in less mature osteoblasts, whereas *Dlx3* is very highly expressed in differentiated osteoblasts and osteocytes, suggesting that *Dlx2* and *Dlx5/6* can stimulate osteoblastic differentiation and that *Dlx3* plays a discrete role in late-stage osteoblast differentiation [32]. The OSX zinc finger protein is a cofactor that binds to Dlx5 to activate the osteoblast differentiation program, and the p53 tumor suppressor can suppress this program by competitive binding to OSX, which thereby diminishes Dlx5 function [41]. Such differentiation can be suppressed by p53. Human DLX proteins play an important role in bone development, and disruption of DLX function underlies the etiology of certain bone/joint diseases. For example, a 4-bp deletion of the *DLX3* gene has been reported in families with tricho-dento-osseous syndrome (TDO), which is characterized by abnormalities involving hair, teeth, and bone development. Transgenic mice harboring such a deletion demonstrated enhanced trabecular bone volume and mineral density, suggesting a novel role for Dlx3 in osteoclast differentiation and bone resorption [42].

*Dlx4* is expressed in the mesenchyme of murine palatal shelves during embryonic development, and a specific mutation in *DLX4* (c.546delG) causes familiar cleft lip and/or palate [43]. Macrodactyly is a congenital disease characterized by overgrowth of soft tissues and bones. RNA-seq analysis has revealed that *DLX5* is upregulated by an activating mutation in the phosphatidylinositol 3-kinase, catalytic alpha gene (*PIK3CA*) in macrodactyly-derived bone marrow mesenchymal stem cells (BMSCs), implying that *DLX5* has contributes to bone overgrowth due to constitutive PI3K/AKT signaling [44]. *DLX* also has a role in chondrocyte proliferation. Chondrocyte hypertrophy is a hallmark of osteoarthritis (OA) pathology. Knockdown of *Dlx5* in BMSCs reduced cell hypertrophy and apoptosis. Overexpression of *DLX5* in human-cartilage-derived mesenchymal progenitors increased the expression of hypertrophy markers and enhanced apoptosis, suggesting that DLX5 is a biomarker of OA changes in human knee joint tissues by contributing to hypertrophy and apoptosis in BMSCs [45]. Utilizing the regulatory features of DLX transcription factors has practical applications. For example, recently DLX was found be able to reprogram somatic cells into induced pluripotent stem cells (iPSCs), with DLX4 being able to functionally replace c-MYC to support efficient reprogramming of human dental pulp cells, in combination with OCT3/4, SOX,2 and KLF4 [46].

## 4. *DLX* Genes in Normal Hematopoiesis

**Figure 3:**
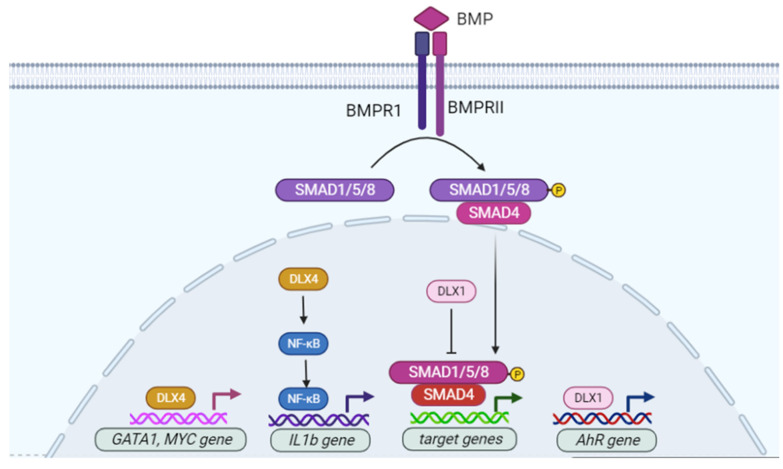
Role of DLX4 in hematopoiesis. DLX1 regulates BMP4 signaling via binding to SMAD4 and inhibiting its transactivation activity. DLX1 also directly regulates the expression of transactivating aryl hydrocarbon receptor (*AhR*) during T/B cell differentiation. DLX4 also enhances NF-κB signaling to promote IL1β production in a megakaryocytic transcriptional program. In addition, DLX4 has been shown to sustain the viability of K562 erythroleukemia cells by promoting the expression of *GATA1* and *MYC*.

Some transcription factors that are essential for the development of the nervous system are also involved in hematopoiesis. For example, *Gata2* knockout mice have severe defects in neurogenesis as well as hematopoiesis [47]. *Dlx* genes have similar dual roles in neural and hematopoietic systems. For instance, *Dlx* genes have been found to be co-expressed with *Bmp4* in some tissues during embryogenesis, and Bmp4 is a TGF-β family member that plays an important role in the differentiation of early mesodermohematogenic cells and hematopoietic stem cells [48,49]. DLX1 interacts with SMAD4 via its homeobox domain, which interferes with the transactivation of SMAD4 (Figure 3). Thus, DLX1 can regulate the function of members of the TGF-β family during hematopoiesis [50]. In the developing thymus, *Dlx1* and *Dlx2* have been detected in thymocytes from 13.5- and 16.5-day-old embryos. Although *Dlx1* knockout mice did not have any discernable developmental defects in either the thymus or thymocyte development, expression of *Dlx1* in neural crest derivatives suggested a potential redundant role in cell migration/migration with other homeobox genes [51].

Actually, *Dlx* genes play a critical role in the development of a subtype of lymphocytes. In the bone marrow of adult mice, *Dlx1, Dlx2*, and predominantly *Dlx3* are transiently expressed in immature Mac-1(lo) NK cells, whereas in mature splenic NK cells, such expression was abolished [52]. The persistent expression of *Dlx* genes leads to functionally immature NK cells arrested at the Mac-1(lo) stage. Moreover, persistent *Dlx1* expression stalls the differentiation of T-cells and B-cells [52]. This occurs at least partially via the transactivating aryl hydrocarbon receptor (AhR), which is a transcription factor essential for the development of some immune cell subsets [53] (Figure 3). Another *DLX* family member, *DLX4*, is expressed in normal hematopoietic cells and human leukemia cell lines with erythroid characteristics. Antisense oligonucleotides targeting *DLX4* have been shown to trigger apoptosis in the human erythroleukemia cell line K562, in connection with a reduction in *GATA1* and *MYC* mRNA levels [54]. During megakaryopoiesis, *DLX4* expression increases, but during erythropoiesis, it decreases. DLX4 induces IL1β production, which turns on NF-κB signaling and potentiates a megakaryocytic transcriptional program. Blocking NF-κB activity reverses this program toward differentiation into erythroid lineages [55] (Figure 3).

## 5. *DLX* Genes in Aberrant Hematopoiesis

T-cell acute lymphoid leukemia (T-ALL) is thought to originate from arrested T-cell progenitors during differentiation. Due largely to specific chromosomal rearrangements, the aberrant expression of certain NKL homeobox genes disrupt T-cell differentiation and give rise to T-ALL. However, the role of NKL homeobox genes is highly context dependent. For example, the *MSX1* gene is normally expressed in common lymphoid progenitors (*CLP*) and remains active in NK cells. Interestingly, *MSX1* behaves as an oncogene in T-ALL but acts as a tumor suppressor gene in NK-cell leukemia [56,57]. Acute myeloid leukemia (AML) frequently has activating mutations in the FMS-like tyrosine kinase-3 gene (*FLT3*), which are a poor prognostic marker. Notably, *DLX1/2* are downstream targets of aberrant FLT3 signaling via the MAPK pathway. Inhibition of FLT3 results in reduced levels of DLX1/2, which in turn enhances TGF-β signaling [58]. In leukemic patients with the t(4;11)(q21;q23) chromosomal translocation, which generates a MLL-AF4 fusion protein (now known as KMT2A-AFF1), the expression of *DLX2, DLX3*, and *DLX4* was diminished; these findings indicate that intact MLL/KMT2A1 normally regulates the expression of these *DLX* family members [59]. In pediatric B-ALL patients with a MLL-AF4 rearrangement, the *DLX3* gene has aberrant CpG methylation, which results in reduced expression of DLX3; in contrast, patients with a TEL-AML1 rearrangement, which has a better prognosis, did not have such methylation [60]. Hypermethylation of the *DLX5* gene occurs frequently in AML and myelodysplastic syndrome (MDS). Such silencing of *DLX5* is associated with a lower rate of complete remission and poorer overall survival, suggesting a tumor suppressing role of DLX5 in AML and MDS [61].

**Figure 4:**
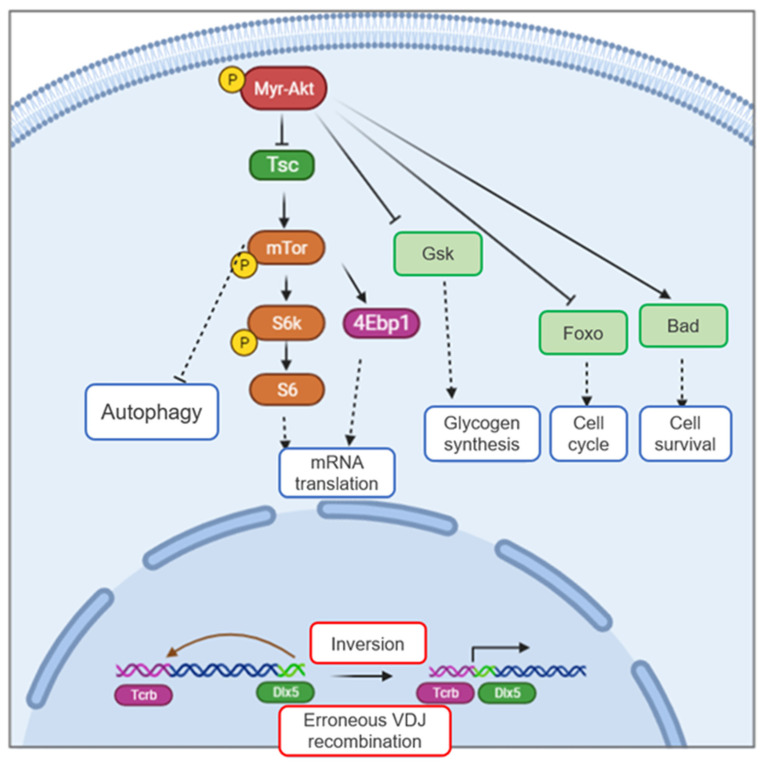
Activation of the *Dlx5* gene triggers T-cell lymphoma. Expression of a constitutively activated form of *Akt2 (MyrAkt2)* in immature mouse thymocytes induces T-cell lymphoma via activation of *Dlx5* gene expression, due to a recurrent chromosome rearrangement with *Tcrb*. Constitutive activation of the Akt pathway promotes survival in cells that undergo aberrant *VDJ* rearrangement and would otherwise undergo apoptosis, whereas the acquisition of T-cell-specific overexpression of *Dlx5* gene provides a proliferative advantage that, together, result in lymphomagenesis.

**Figure 5:**
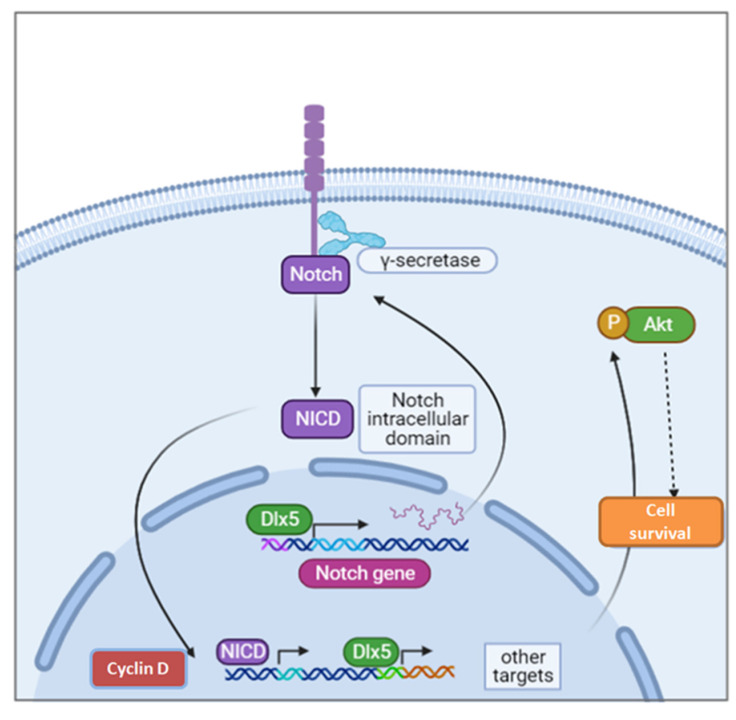
Dlx5 directly induces T-cell lymphoma by activating *Notch*. A transgenic mouse model expressing *Dlx5* gene under the control of a Lck promoter develops a high incidence of T-cell lymphomas with overexpression of Notch genes, *Notch1* and *Notch3*. These lymphoma cells were very sensitive to γ-secretase inhibitors and exhibited upregulation of Akt signaling and upregulation of Cyclin D.

Transgenic mice expressing a constitutive activation of the *Akt2* oncogene specifically in immature T-cells, *Lck-MyrAkt2* mice, develop a high rate of spontaneous thymic lymphomas, including some founders that acquire a recurring chromosomal inversion that juxtaposes the enhancer of the T-cell receptor-β (*Tcrb*) locus and the *Dlx5/6* bigene, thereby, resulting in overexpression of Dlx5 and, to a lesser extent, Dlx6 [62] (Figure 4). Similar to other *Dlx* family members, mouse *Dlx5* and human *DLX5* are not expressed in mature thymocytes [52,62]. However, *DLX5* mRNA, but not *DLX6* mRNA, was abundantly expressed in three of seven human T-cell lymphomas we tested [62]. Subsequent transgenic mouse experiments revealed that forced expression of *Dlx5* in immature T-cells using a Lck promoter (*Lck-Dlx5* mice), also induced thymic lymphomas [63]. Whole transcriptome analysis showed that these thymic lymphomas consistently showed upregulation of Notch1 and Notch3, and in vitro experiments revealed that these lymphoma cells were highly sensitive to Notch inhibitors [63]. Additionally, Dlx5 was found to directly bind to the regulatory elements of the *Notch1* and *Notch3* genes, as revealed by ChIP-seq analysis (Figure 5), and Dlx5 was able to transactivate luciferase expression by binding to these elements in vitro [63].

**Figure 6:**
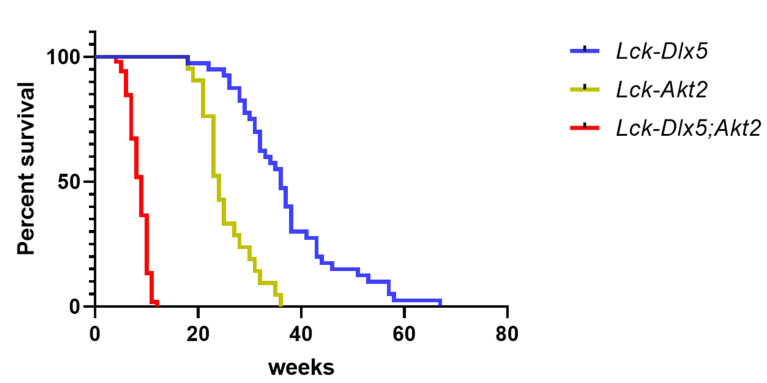
Survival curve of *Lck*-*Dlx5* mice with T-cell lymphomas as compared with *Lck-MyrAkt2* and double transgenic *Lck-MyrAkt2; Dlx5* mice. The median survival was 24 weeks in *Lck-Dlx5* mice, 39 weeks in Lck-Myr*Akt2* mice, and 10 weeks in *Lck-MyrAkt2;Dlx5* mice.

Other NKL members such as MSX2, TLX1, and NKX2-5 can also upregulate *Notch3* by interacting with NOTCH pathway repressors [10]. Moreover, Akt signaling and c-Myc levels are consistently elevated in Dlx5-induced lymphomas, and pharmacological inhibition of Akt and c-Myc triggers these lymphoma cells to undergo apoptosis [64]. These observations suggests that prolonged expression of Dlx5 in progenitor T-cells trigger lymphomagenesis via the activation of oncogenic pathways commonly involved in T-lymphomagenesis, including Notch, Myc, and Akt. On the one hand, activation of these signaling pathways promotes cell survival and inhibit apoptosis when Tcr rearrangements go awry. On the other hand, frequent upregulation of Wnt signaling has also been reported in pediatric T-ALL [65], and an activating mutation of the β-catenin gene, *Ctnnb1*, has been shown to induce T-ALL in mice without Notch upregulation [66]. The medium survival of these *Ctnnb1*-mutant mice was 14 weeks as compared with 24 weeks in *Lck-MyrAkt2* mice and 39 weeks in *Lck-Dlx5* mice. This suggests that *Ctnnb1* behaves as a strong oncogene in a T-cell transgene setting, whereas active Akt2 is less oncogenic, and Dlx5 is weakly oncogenic [67]. Interestingly, however, the median survival of *Lck-MyrAkt2;Dlx5* double transgenic mice was 10 weeks, suggesting a synergistic effect between Akt and Dlx5 (Figure 6) [67]. This synergism is likely due to the fact that β-catenin is strongly expressed and resides in nucleus of the T-cell lymphomas from the *Lck-MyrAkt2;Dlx5* double transgenic mice.

**Figure 7:**
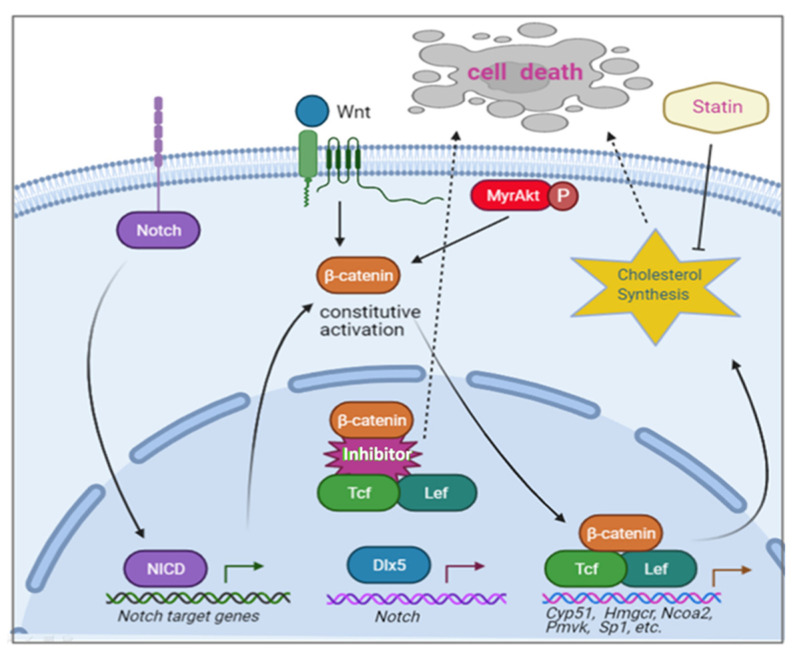
Dlx5 cooperates with activated Akt (MyrAkt) to accelerate T-cell lymphoma in a GEM model. The Wnt pathway was activated when mouse T-cells expressed both *Dlx5* and *MyrAkt2* transgenes. Inhibition of the β-catenin/Tcf complex resulted in apoptosis. β-catenin/Tcf directly transactivates several key components in the cholesterol synthesis pathway, such as *Cyp51*, *Hmgcr*, and *Ncoa2*. The augmented cholesterol synthesis at least partially underlies the oncogenic role of Wnt signaling, as statin treatment triggered cell death in these cells.

Despite the multiple genes and pathways activated in these lymphomas, Wnt signaling appeared to be the key driver, because inhibition of the Wnt pathway triggered rapid cell death [67]. Whether Dlx5 can directly bind to the *Ctnnb1* locus to increase its expression when the cellular context is changed by the addition of Akt hyperactivation is an intriguing question. Further study by RNA-seq analysis has demonstrated that the cholesterol biosynthesis pathway is highly upregulated in lymphoma cells from *Lck-MyrAkt2;Dlx5* mice. The β-catenin/Tcf complex directly binds to genes encoding key members of this pathway, such as** *Cyp51*, *Hmgcr*, *Ncoa2*, *Pmvk*, *Sp1*, *Srebf1*, *Srebf2*, *Tbl1x*, and *Tbl1xr1*. Moreover, statin and other cholesterol inhibitors were shown to effectively limit the proliferation of lymphoma cells from *Lck-MyrAkt2;Dlx5* mice at low concentrations and cause cell death at higher concentrations [67] (Figure 7).

## 6. Involvement of *DLX* Genes in Other Malignancies

Normal cells cannot proliferate infinitely due to telomere erosion, which forces cells to enter a state of replicative senescence by activating ATM/p53 signaling. Interestingly, DLX2 expression has been shown to result in a prolonged replicative life span by diminishing protein components of the TTI1/TTI2/TEL2 complex [68]. This complex is essential for the proper folding and stabilization of ATM and other members of the PI3K-related kinase family kinase (PIKK), resulting in weakened ATM/p53 signaling and senescence bypass. The investigators also found that overexpression of DLX2 displayed a mutually exclusive relationship with p53 defects in cancer patients [68]. In related work, Yilmaz and colleagues have shown that DLX2 protect against transforming growth factor β (TGFβ)-induced cell-cycle arrest and apoptosis [69]. TGFβ acts as a tumor suppressor by inhibiting cell cycle progression during the early stages of carcinogenesis, whereas it shows tumor promoting activity at later stages. The investigators found that Dlx2 exerted important functions in flipping this switch, doing so in part by directly suppressing the transcription of TGFβ receptor II and the cell cycle inhibitor p21 (CDKN1A), diminishing SMAD signaling, enhancing c-MYC transcription, and increasing EGFR signaling. Dlx2 expression was also found to promote tumor invasion and metastasis [69].

TGFβ signaling is known to induce epithelial to mesenchymal transition (EMT) via upregulation of SNAIL [70]. Moreover, overexpression of Dlx2 induces the expression of SNAIL, and knockdown of *Dlx2* blocks TGFβ-induced EMT [71]. Dlx2 also induces the expression of the glutamine metabolism enzyme glutaminase (GLS1), and knockdown of *Dlx2* reduces glutamine metabolism, which results in SNAIL expression [72]. In glioblastoma multiforme (GBM) patients, high levels of DLX2 have been associated with a poor survival outlook, and knockdown of *DLX2* in GBM cells reduced cyclin D1 expression [73]. However, the role of DLX2 appears to be cancer-type dependent. For example, during glucose deprivation-driven metabolic stress in breast cancer cells, DLX2 was induced by reactive oxygen species, and knockdown of *DLX2* protected cells from necrosis [74]. Moreover, expression of DLX2 and DLX5 have been reported to be mutually exclusive in breast cancer, with DLX2 expression being significantly correlated with a favorable prognosis, whereas DLX5 was associated with metastasis [75].

DLX4 is also involved in switching TGFβ signaling from tumor suppressing to tumor promoting. DLX4 has been shown to inhibit TGFβ-induced expression of p15(Ink4b) and p21 by binding to and inhibiting Smad4 from forming complexes with Smad2 and Smad3 [76]. Moreover, the same investigation revealed that expression of DLX4 stimulated the expression of c-Myc independently of TGFβ/Smad signaling. In breast cancer, patients whose tumors express high levels of DLX4 respond poorly to topoisomerase II (TOP2)-targeting chemotherapy, which kills tumor cells by inducing DNA double-strand breaks (DSB) [77]. Mechanistically, DLX4 was found to interact with Ku proteins to promote DNA-dependent protein kinase activity and end-joining repair of DSBs, thereby, reducing sensitivity of tumor cells to TOP2 poisons. In other work, DLX4 was shown to promote EMT in breast cancer cells through TWIST [78]. DLX4 directly bound to the *TWIST* gene promoter to regulate its expression, and DLX4 overexpression enhanced expression of TWIST. Furthermore, knockdown of *DLX4* decreased TWIST expression, resulting in reduced migration ability of breast cancer cell lines. In one study, the gene copy number of *DLX4* was elevated in about 22% of primary breast cancer and 24% of the cancers with sentinel lymph node (SLN) metastasis [79]. DLX4 has also been documented to promote expression of iNOS via binding to STAT1, and the elevated levels of this enzyme triggered angiogenesis by producing nitric oxide [80]. High DLX4 expression in ovarian cancer strongly correlated with elevated levels of iNOS and poor survival. Furthermore, expression of DLX4 in ovarian cancer cells potentiated endothelial cell proliferation in vitro and microvessel formation in xenograft tumors [80]. DLX4 also induced the expression of CD44 in ovarian cancer cells, and inhibition of CD44 abolished DLX4′s ability of DLX4 to promote tumor-mesothelial cell interactions in these cells [81]. Induction of CD44 by DLX4 was connected with enhanced NF-κB activity, which was stimulated by IL-1β, a transcriptional target of DLX4.

**Figure 8:**
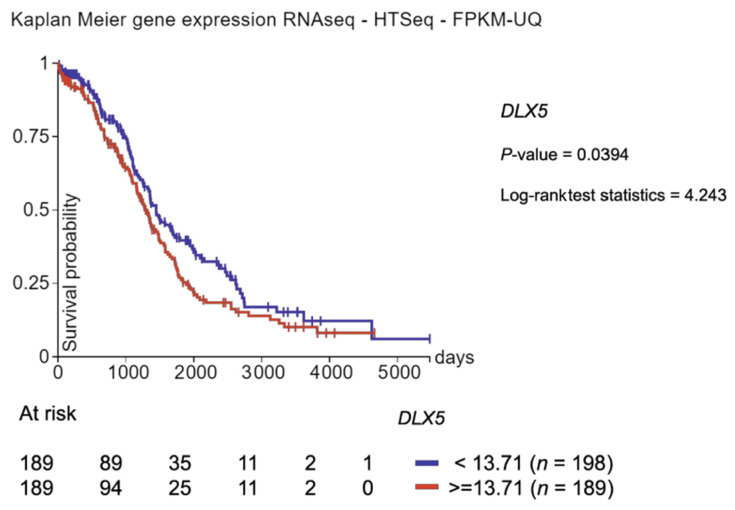
*DLX5* is a poor prognosis marker in ovarian cancer. The RNA expression level of *DLX5* in The Cancer Genome Atlas (TCGA) indicate that higher expression of *DLX5* transcripts is associated with poorer survival in ovarian patients. HTSeq-FPKM-UQ = High Throughput Sequencing-Fragments Per Kilobase of transcript per Million mapped reads-Upper Quartile.

**Figure 9:**
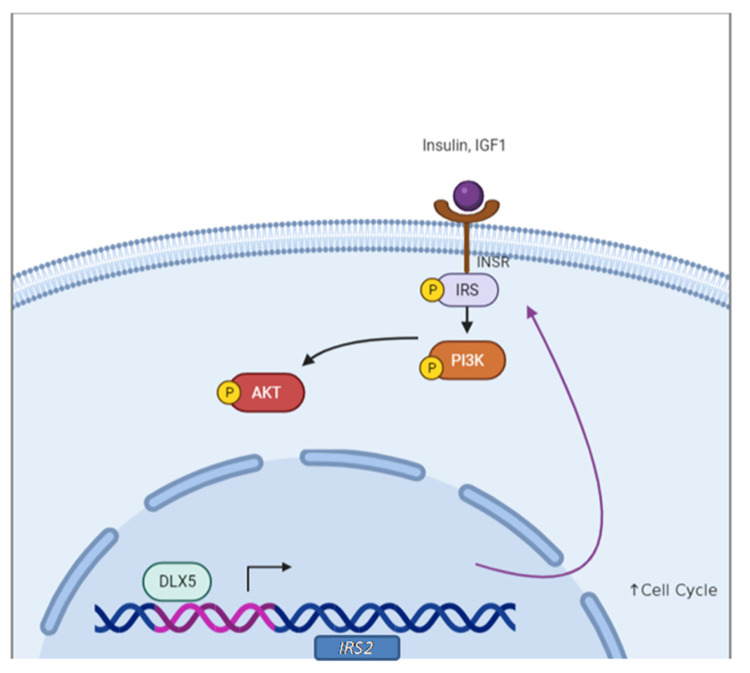
DLX5 upregulates *IRS2* expression to enhance AKT signaling. In human ovarian cancer, DLX5 can directly bind to the *IRS2* promoter and increase its expression. The resulting elevated IRS2 levels, in turn, augment AKT signaling, which is essential for tumor cell survival.

The *MYC* gene has been shown to be a transcriptional target of DLX5 [82]. DLX5 binds to the *MYC* promoter and activates MYC expression in vitro, as shown by a *MYC* promoter assay in HEK 293 cells. Moreover, overexpression of DLX5 results in increased cell proliferation by upregulating MYC. In a screen of the NCI 60 cancer cell line panel, DLX5 was frequently upregulated in cell lines derived from several tumor types, including ovarian cancer [83], and data from The Cancer Genome Atlas indicate that DLX5 is a poor prognosis marker in ovarian cancer (Figure 8). We found that overexpression of DLX5 promoted ovarian cancer cell proliferation by augmenting IRS-2/AKT signaling [83]. DLX5 potentiated AKT signaling to promote tumor cell proliferation, and knockdown of *DLX5* reduced cell viability and downregulated IRS-2 and AKT phosphorylation. Moreover, DLX5 was found to directly bind the *IRS2* promoter and regulate *IRS2* expression (Figure 9) [83].

*DLX5* and other homeobox genes such as *HOXA* have been shown to be methylated in early stage lung cancers [84]. In a study of human non-small cell lung cancer (NSCLC), *DLX5* was determined to be activated by KDM4A-mediated demethylation, and in turn, DLX5 induced the expression of MYC and β-catenin, thereby, promoting proliferation and metastasis [85]. Kato et al. found that the expression level of DLX5 protein significantly correlated with tumor size and poorer prognosis in NSCLC patients [86]. Moreover, knockdown of *DLX5* with small interfering RNAs markedly suppressed the proliferation of NSCLC cells [86]. The homeobox genes *DLX2*, *DLX5,* and *HOXB2* were each overexpressed in a subgroup of small cell lung cancer cell lines [87]. In endometrial carcinomas, DLX5 was upregulated in tumors of the endometrioid subtype, but not in those with papillary serous features [88]. Recently, miRNA have been implicated in regulating *DLX* gene function in carcinogenesis. For example, in prostate cancer, miR-489-3p directly targets *DLX1* and downregulates its expression; overexpression of miR-489-3p was shown to induce apoptosis [89]. In hepatocellular carcinoma, miR-122 was reported to be downregulated; miR-122 was able to bind to the 3′UTR of *DLX4* mRNA, resulting in down regulated expression of DLX4 protein [90].

## 7. Conclusions

The Antennapedia (ANTP) class makes up the largest of the homeobox gene classes in animal genomes [2]. Hox and ANTP-like homeobox gene classes play key roles in the development of bilaterians, animals with bilateral symmetry as an embryo. This gene group was fundamental to the origin of metazoan life on earth [91]. As a subgroup of such ancient master regulators, *DLX* homeobox family genes play important roles not only in embryogenesis primarily involving neuronal differentiation, cranial and limb development, but also in postnatal homeostasis, such as hematopoiesis and, when dysregulated, oncogenesis. DLX family members are implicated in crosstalk with other signaling and developmental pathways in tissue specific microenvironments. Disturbances of DLX proteins by genomic alterations either at the enhancer level or within the coding region result in congenital disorders such as tricho-dento-osseous syndrome and split-hand/foot malformation. Dysregulation of *DLX* gene expression is also involved in various types of cancers, including leukemias and lymphomas. With a deeper understanding of DLX’s role in human disease, future therapeutic approaches aimed at correcting aberrant *DLX* gene expression, via pharmacological or biological means, hold promise for alleviating these diseases.

## Data Availability

Not applicable.
